# A surgical strategy-based nomogram for primary pulmonary mucoepidermoid carcinoma

**DOI:** 10.1007/s12672-025-03117-7

**Published:** 2025-08-15

**Authors:** Lei Yu, Yanling Peng, Shan Lan, Lu Yang

**Affiliations:** 1 Department of Oncology, Suining Central Hospital, Suining, Sichuan China; 2 The First People’s Hospital of Guangyuan, Guangyuan City, China

**Keywords:** Primary pulmonary mucoepidermoid carcinoma, Nomogram model, Rare disease management, Overall survival

## Abstract

**Background:**

Primary pulmonary mucoepidermoid carcinoma (PMEC) is a rare malignancy with no standardized prognostic tools. This study aimed to investigate whether specific surgical approaches significantly impact survival outcomes and to develop an accurate predictive nomogram incorporating surgical variables.

**Methods:**

Using the SEER database (2004–2020), we identified 225 PMEC patients. Independent prognostic factors were selected via Cox regression (*P* < 0.05). A nomogram was developed and validated using time-dependent ROC curves, calibration plots, and decision curve analysis (DCA).

**Results:**

We analyzed 225 PMEC cases, predominantly affecting patients aged 30–69 years with slight male predominance. Most tumors (30–52 mm) were early-stage with favorable prognosis; only 11.01% presented as Stage IV (3-year OS: 18.5%, median survival: 8.5 months). The nomogram (incorporating age, tumor size, N/M stage, surgery, and chemotherapy) demonstrated excellent discrimination (AUC > 0.9) and calibration, with pneumonectomy plus lymph node dissection showing the best surgical outcomes.

**Conclusion:**

Our nomogram provides a clinically useful tool for PMEC prognostication, validating that lymph node dissection during lobectomy significantly improves survival. It may guide individualized surgical decisions for this rare malignancy.

## Introduction

Primary pulmonary mucoepidermoid carcinoma (PMEC) is a rare malignancy, accounting for only 0.1–0.2% of all lung cancers, yet its clinical management remains poorly standardized due to limited epidemiological and prognostic data [1–3]. Unlike its salivary gland counterpart, PMEC lacks distinctive clinical or radiological features, often mimicking common lung cancers, which complicates diagnosis and delays treatment [4–8]. Histologically, PMEC’s heterogeneous composition of squamous, mucous, and intermediate cells further contributes to diagnostic challenges, particularly in small biopsies or poorly differentiated cases [9]. The prognosis of PMEC is intricately tied to the stage of the disease at the time of detection. Early diagnosis often portends a favorable prognosis, whereas late-stage patients face a dim prognosis, with a five-year overall survival (OS) rate plummeting to 23.8% [10], underscoring the need for robust risk stratification tools.

Nomograms have emerged as valuable instruments for personalized prognosis in oncology, integrating clinical, pathological, and therapeutic variables into accessible predictive models [11]. While previous PMEC studies identified factors such as age, tumor size, and TNM stage as prognostic markers [12], the impact of surgical approaches—a critical determinant of survival—has been understudied. Current literature suggests lobectomy as the primary intervention [13], but comparative analyses of techniques (e.g., pneumonectomy, lymph node dissection) are lacking. Moreover, chemotherapy’s role remains controversial, with anecdotal evidence supporting EGFR inhibitors in selected cases [14, 15].

To address these gaps, we hypothesized that: (1) surgical modalities significantly impact survival outcomes in PMEC, and (2) a prognostic nomogram integrating these surgical factors with clinical/pathological variables could enhance treatment personalization.

## Materials and methods

### Data sources

**Fig. 1:**
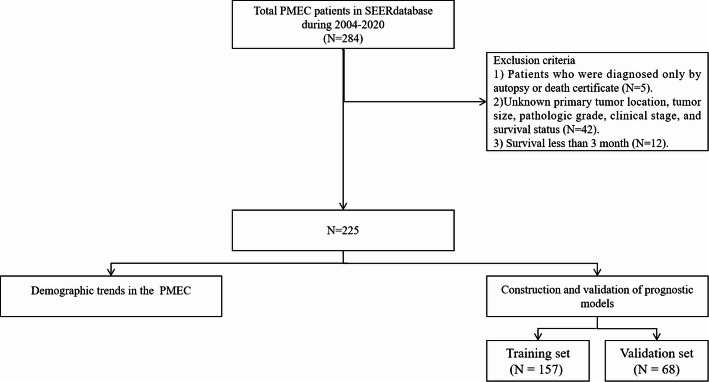
Flowchart of the study. SEER, surveillance, epidemiology and end results

This study primarily draws its data from the Surveillance, Epidemiology, and End Results (SEER) Database [16], which compiles demographic, clinicopathological, and survival data on cancer patients. The data, accumulated from 17 established cancer registries covering roughly one-third of the U.S. population, was accessed and extracted specifically for PMEC cases following authorization, utilizing the SEER*Stat software (version 8.4.2, available at https://seer.cancer.gov/). Inclusion criteria for our dataset were meticulously defined as follows: 1. Cases diagnosed within the period spanning from 2004 to 2020;2. Assignment of the International Classification of Diseases for Oncology (ICD-O) histological code 8430/3, indicative of mucoepidermoid carcinoma;3. Classification as the first primary tumor;4. Availability of data for the following inclusion variables: baseline demographics (age, sex, race, vital status, and survival months), tumor characteristics (anatomical location, tumor size, laterality, differentiation grade, T stage, N stage, M stage), and treatment modalities employed (surgery, radiation therapy, chemotherapy).Exclusion criteria were stringently applied to ensure data integrity, eliminating cases that:1. Were solely diagnosed via autopsy or death certificate;2. Presented with missing information crucial for analysis;3. Demonstrated a survival duration of less than three months post-diagnosis (Fig. 1).

### Construction and validation of the prognostic nomogram model

The X-title method (Yale University School of Medicine, version 4.1.2) was employed to ascertain the optimal cutoff value for the continuous variable (tumor size) due to its ability to optimize survival-based cutoffs through recursive partitioning, which maximizes statistical relevance while preserving continuous data integrity. Widely validated in studies, this approach outperforms arbitrary thresholds by identifying clinically meaningful size boundaries [17, 18]. Following the organization of the PMEC patient data, the sample.split function in the caTools function package of R software was used to randomly divide all patients into training and validation cohorts in a 7:3 ratio. The random seed was set at 123 to guarantee reproducibility. The former was utilized to develop the nomogram, while the latter served for its subsequent validation. Initiating the statistical analysis within the training set, univariate regression analyses were conducted, with variables exhibiting *P* < 0.1 being advanced to the multivariate regression analysis. This rigorous process facilitated the identification of independent risk factors influencing the prognosis of PMEC. These selected variables then formed the foundation for constructing the prognostic nomogram. To comprehensively evaluate the nomogram’s predictive performance, we conducted rigorous validation analyses across both training and validation cohorts. First, time-dependent receiver operating characteristic (ROC) curve analysis quantified the model’s discriminative ability, with the area under the curve (AUC) providing a standardized metric of predictive accuracy. We further assessed prediction reliability through calibration curve analysis, which graphically compared nomogram-predicted survival probabilities against observed outcomes. Finally, decision curve analysis (DCA) was employed to determine the clinical applicability of the model by calculating net benefit across a range of clinically relevant probability thresholds. This tripartite validation framework (ROC analysis for discrimination, calibration curves for accuracy, and DCA for clinical utility) was systematically applied to both the training and validation datasets to ensure robust performance assessment.

### Statistical analysis

This study harnessed the capabilities of SPSS version 26.0 and R software (version 4.3.1) for comprehensive data assessments. Categorical variables were expressed in terms of frequencies and percentages, with comparisons among these variables executed through chi-square tests or Fisher’s exact test where appropriate. All statistical tests adhered to a two-tailed design, and a P-value below 0.05 was deemed statistically significant.

## Results

**Fig. 2:**
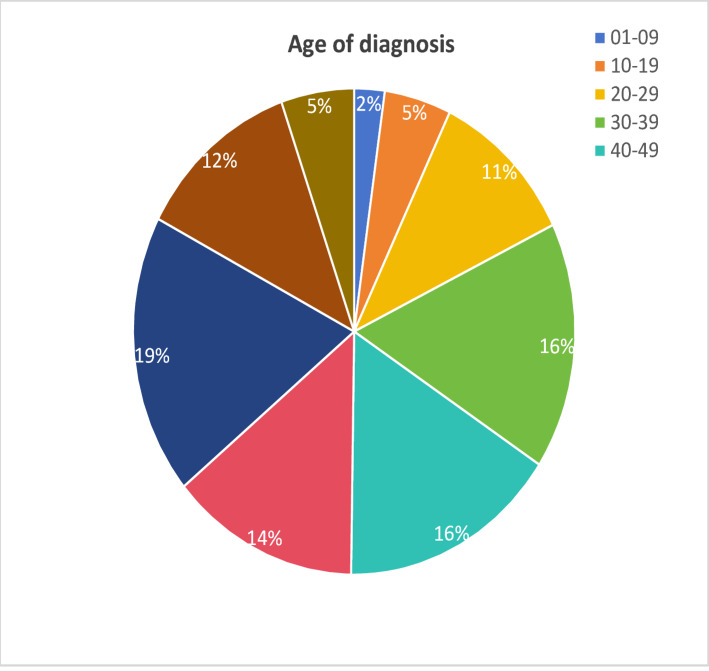
Age of diagnosis

Between 2004 and 2020, under stringent inclusion and exclusion criteria, a total of 225 PMEC patients were enrolled in our study. In analyzing the diagnostic ages of PMEC patients, we found that the age groups 60–69 years (18.7%), 30–39 years (16.44%), and 40–49 years (16%) represent peak incidence periods for PMEC (Fig. 2). Gender distribution indicated a slight male preponderance, with males constituting 55.11% of cases compared to 44.89% females, primarily affecting the Caucasian population.Regarding anatomical location, PMEC lesions predominantly affected the upper and lower lung lobes, with only 12% of cases involving the middle lobe, exhibiting no marked lateral preference between the right and left lungs. Using the X-title software, PMEC patients’ tumor sizes were stratified into three groups based on the optimal cutoff point: <30 mm, 30–52 mm, and > 52 mm, revealing that over half of the patients (56%) had tumors measuring less than 30 mm. Most PMEC cases demonstrated favorable histological differentiation (64.88%), whereas poorly differentiated and undifferentiated cases accounted for a minority at 14.67%.Metastatic patterns showed that 18.67% of patients experienced lymph node metastasis, and 10.67% developed distant metastases. The most frequent sites of distant metastasis were bone (*N* = 10), lung (*N* = 8), and brain (*N* = 6). In terms of treatment strategies, surgery emerged as the principal intervention, with an impressively high 86.68% of patients undergoing surgical procedures. Surgical interventions varied, with “lobectomy combined with mediastinal lymph node dissection” being the most prevalent approach at 52.89%, followed by simple lobectomy at 22.67%, and a more extensive lung resection performed in 11% of patients due to specific clinical necessities (Table 1).

**Fig. 3:**
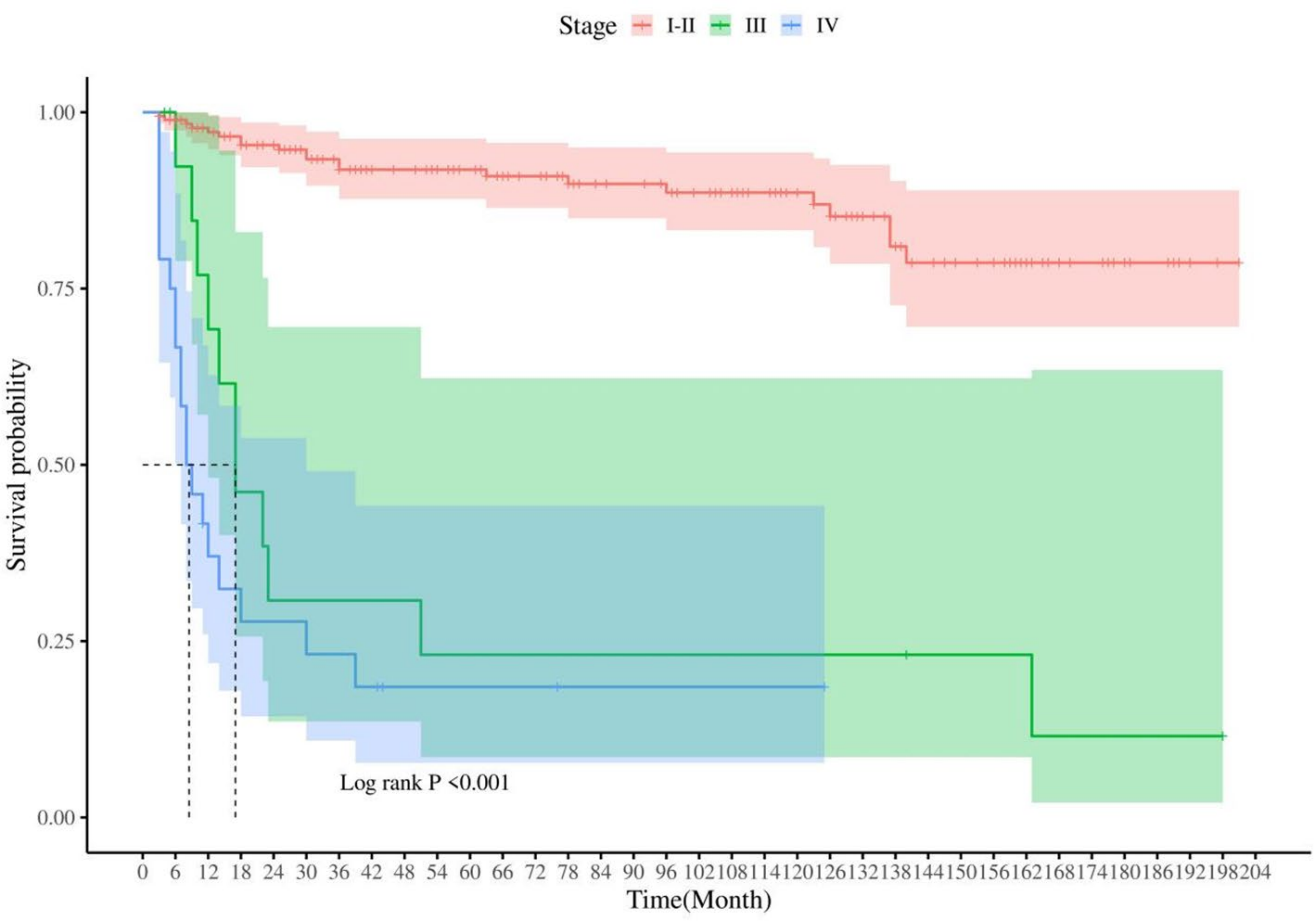
Survival time of PMEC patients at different stages

Prognostically, PMEC demonstrated a more favorable early survival profile compared to common lung cancers. Specifically, stage I-II patients achieved a 5-year OS rate of 90.9%. However, this figure plummeted to 23.1% for 3-year OS in stage III patients, with a median survival period of 17 months. For late-stage IV patients, the 3-year OS rate was 18.5%, and their median survival time was truncated to merely 8.5 months (Fig. 3).

**Table 1:** Basic demographic and clinical characteristics of PMEC patients in the training and validation set

Variables	Total (*n* = 225)	Validation set (*n* = 68)	Training set (*n* = 157)	Statistic	*P*
Primary Site, n(%)				χ²=0.08	0.961
Lower	106 (47.11)	33 (48.53)	73 (46.50)		
Middle	27 (12.00)	8 (11.76)	19 (12.10)		
Upper	92 (40.89)	27 (39.71)	65 (41.40)		
Race, n(%)				χ²=3.66	0.160
Black	23 (10.22)	3 (4.41)	20 (12.74)		
Other	30 (13.33)	9 (13.24)	21 (13.38)		
White	172 (76.44)	56 (82.35)	116 (73.89)		
Age, n(%)				χ²=0.02	0.885
< 60	144 (64.00)	44 (64.71)	100 (63.69)		
>= 60	81 (36.00)	24 (35.29)	57 (36.31)		
Sex, n(%)				χ²=0.52	0.470
Female	101 (44.89)	33 (48.53)	68 (43.31)		
Male	124 (55.11)	35 (51.47)	89 (56.69)		
Grade, n (%)				χ²=0.12	0.989
1	64 (28.44)	20 (29.41)	44 (28.03)		
2	82 (36.44)	25 (36.76)	57 (36.31)		
3	33 (14.67)	10 (14.71)	23 (14.65)		
Unknown	46 (20.44)	13 (19.12)	33 (21.02)		
Tumor size, n(%)				χ²=1.60	0.449
< 30 mm	126 (56.00)	42 (61.76)	84 (53.50)		
30–52 mm	72 (32.00)	20 (29.41)	52 (33.12)		
>52 mm	27 (12.00)	6 (8.82)	21 (13.38)		
Laterality, n(%)				χ²=0.34	0.560
Left	96 (42.67)	31 (45.59)	65 (41.40)		
Right	129 (57.33)	37 (54.41)	92 (58.60)		
Stage, n (%)				χ²=0.58	0.902
I	149 (68.35)	46 (70.77)	103 (67.32)		
II	30 (13.76)	8 (12.31)	22 (14.38)		
III	15 (6.88)	5 (7.69)	10 (6.54)		
IV	24 (11.01)	6 (9.23)	18 (11.76)		
Stage T, n(%)				–	0.157
T1	116 (51.56)	41 (60.29)	75 (47.77)		
T2	79 (35.11)	17 (25.00)	62 (39.49)		
T3	15 (6.67)	4 (5.88)	11 (7.01)		
T4	15 (6.67)	6 (8.82)	9 (5.73)		
Stage N, n(%)				–	0.791
N0	183 (81.33)	55 (80.88)	128 (81.53)		
N1	16 (7.11)	5 (7.35)	11 (7.01)		
N2	19 (8.44)	7 (10.29)	12 (7.64)		
N3	7 (3.11)	1 (1.47)	6 (3.82)		
Stage M, n(%)				χ²=0.35	0.556
M0	201 (89.33)	62 (91.18)	139 (88.54)		
M1	24 (10.67)	6 (8.82)	18 (11.46)		
Surg Style, n(%)				χ²=4.17	0.244
Lobectomy	51 (22.67)	19 (27.94)	32 (20.38)		
Lobectomy + MLN	119 (52.89)	33 (48.53)	86 (54.78)		
Pneumonectomy	25 (11.11)	10 (14.71)	15 (9.55)		
None	30 (13.33)	6 (8.82)	24 (15.29)		
Radiation, n(%)				χ²=0.00	0.949
None/Unknown	199 (88.44)	60 (88.24)	139 (88.54)		
Yes	26 (11.56)	8 (11.76)	18 (11.46)		
Chemotherapy, n(%)				χ²=0.15	0.697
No/Unknown	199 (88.44)	61 (89.71)	138 (87.90)		
Yes	26 (11.56)	7 (10.29)	19 (12.10)		

χ²: Chi-square test, -: Fisher exact Lobectomy + MLN: Lobectomy WITH mediastinal lymph node dissection pneumonectomy: complete pneumonectomy, sleeve pneumonectomy, standard pneumonectomy, total pneumonectomy, resection of whole lung

### Independent prognostic factors for OS in PMEC patients

In this study, all patients were randomly allocated to a training set (*N* = 157) and a validation set (*N* = 68), with no statistically significant differences observed in their baseline characteristics (Table 1). To gain deeper insights into the determinants of PMEC prognosis, we conducted univariate and multivariate Cox regression analyses within the training cohort.Univariate Cox regression analyses identified several factors significantly associated with the OS of PMEC patients at the time of diagnosis, including age, tumor size, histological grading, T stage, N stage, M stage, receipt of surgical treatment, radiotherapy, and chemotherapy (all *P* < 0.05) (Table 2). In order to elucidate the independent effects of these variables, we proceeded with a multivariate stepwise regression analysis, incorporating variables with P-values less than 0.1 from the univariate analysis. The results highlighted age, tumor size, N stage, M stage, surgical intervention, and chemotherapy administration as independently predictive of OS in PMEC patients (Table 2). Of particular note, several factors emerged as harbingers of poor prognosis in PMEC: patients aged over 60 years, those with tumors measuring 30–52 mm in diameter, the presence of lymph node and distant metastases, as well as the absence of surgical intervention and chemotherapy, were all identified as independent risk factors for adverse outcomes in PMEC.

**Table 2:** Results of univariate Cox regression and multivariate Cox regression analysis

Variables	Uni-Cox		Mul-Cox	
	*P*	HR (95%CI)	*P*	HR (95%CI)
Sex				
Female		1.00 (Reference)		
Male	0.052	0.53 (0.28 ~ 1.01)		
Age				
< 60		1.00 (Reference)		1.00 (Reference)
>= 60	< 0.001	11.10 (4.86 ~ 25.36)	< 0.001	23.52 (6.68 ~ 82.83)
Race				
Black		1.00 (Reference)		
Other	0.249	0.28 (0.03 ~ 2.47)		
White	0.342	1.66 (0.59 ~ 4.68)		
Primary Site				
Lower		1.00 (Reference)		
Middle	0.791	0.87 (0.32 ~ 2.36)		
Upper	0.853	1.07 (0.54 ~ 2.12)		
Laterality				
Left		1.00 (Reference)		
Right	0.769	0.91 (0.48 ~ 1.72)		
Tumor size				
<30		1.00 (Reference)		1.00 (Reference)
30–52	0.031	2.43 (1.09 ~ 5.45)	0.012	3.37 (1.31 ~ 8.67)
>52	< 0.001	8.47 (3.82 ~ 18.77)	0.563	1.49 (0.39 ~ 5.77)
Grade				
1		1.00 (Reference)		
2	0.586	0.71 (0.20 ~ 2.45)		
3	< 0.001	11.84 (4.13 ~ 33.90)		
Unknown	< 0.001	10.26 (3.57 ~ 29.49)		
Stage T				
T1		1.00 (Reference)		
T2	0.198	1.66 (0.77 ~ 3.59)		
T3	< 0.001	7.95 (3.08 ~ 20.51)		
T4	< 0.001	6.83 (2.38 ~ 19.62)		
Stage N				
N0		1.00 (Reference)		1.00 (Reference)
N1	0.029	3.36 (1.13 ~ 9.98)	0.049	4.15 (1.01 ~ 17.10)
N2	< 0.001	14.69 (6.90 ~ 31.30)	0.086	4.17 (0.82 ~ 21.36)
N3	< 0.001	12.95 (4.24 ~ 39.62)	< 0.001	43.73 (5.35 ~ 357.40)
Stage M				
M0		1.00 (Reference)		1.00 (Reference)
M1	< 0.001	13.59 (6.56 ~ 28.16)	0.004	4.33 (1.59 ~ 11.80)
Surg Style				
Lobectomy		1.00 (Reference)		1.00 (Reference)
Lobectomy + MLN	0.014	0.32 (0.13 ~ 0.79)	0.008	0.26 (0.10 ~ 0.70)
Pneumonectomy	0.493	0.64 (0.18 ~ 2.29)	0.207	0.42 (0.11 ~ 1.62)
None	< 0.001	4.14 (1.90 ~ 9.03)	0.975	0.98 (0.32 ~ 3.05)
Radiation				
None/Unknown		1.00 (Reference)		
Yes	< 0.001	7.59 (3.76 ~ 15.31)		
Chemotherapy				
No/Unknown		1.00 (Reference)		1.00 (Reference)
Yes	< 0.001	6.43 (3.24 ~ 12.76)	0.029	0.29 (0.10 ~ 0.88)

*HR* Hazards Ratio, *CI* Confidence Interval Lobectomy + MLN: Lobectomy WITH mediastinal lymph node dissection pneumonectomy: complete pneumonectomy, sleeve pneumonectomy, standard pneumonectomy, total pneumonectomy, resection of whole lung

### Construction and validation of a nomogram model for OS in PMEC patients

**Fig. 4:**
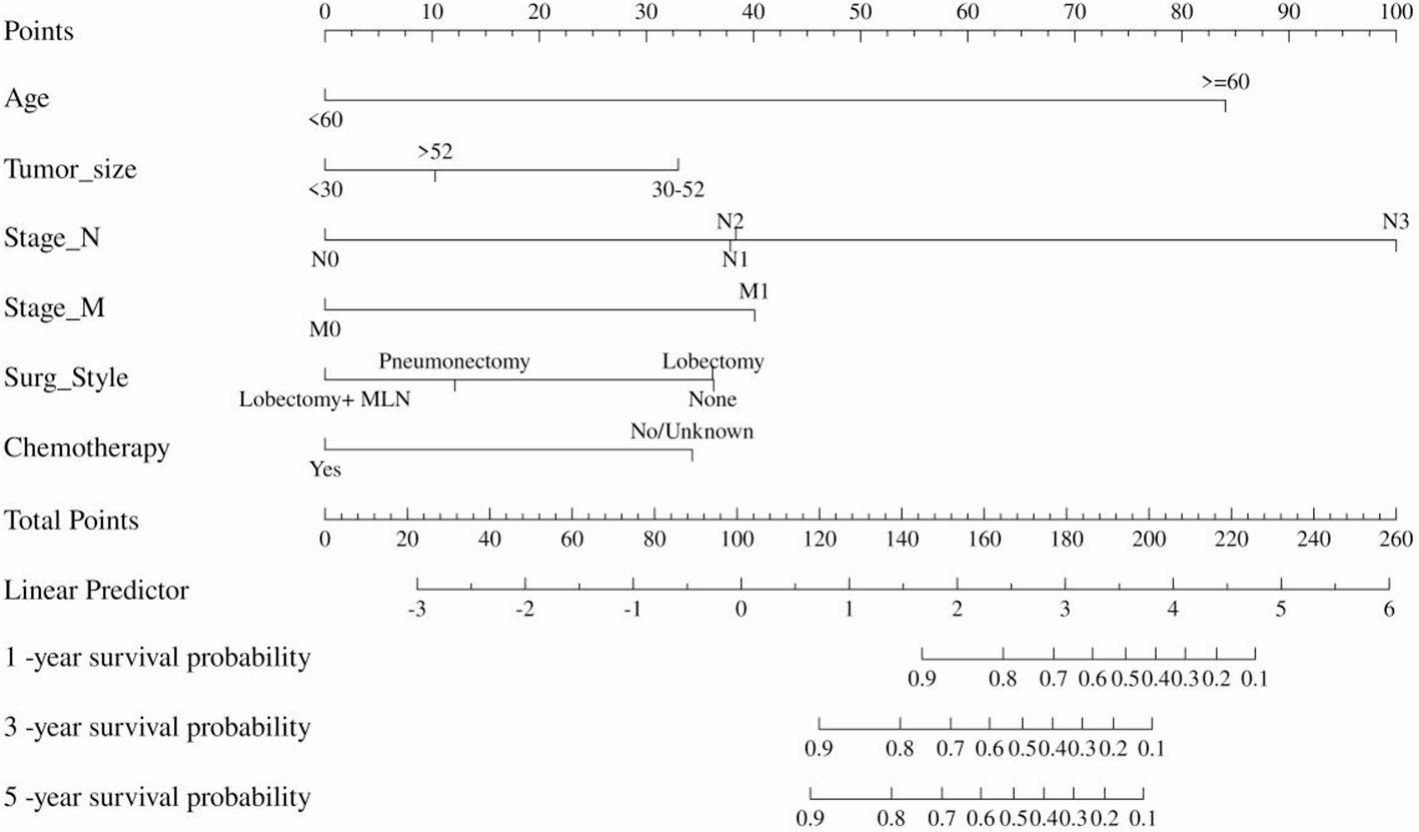
Nomogram for predicting 1-, 3-, and 5-year OS of patients with PMEC.Lobectomy + MLN: Lobectomy WITH mediastinal lymph node dissection; Pneumonectomy; complete pneumonectomy,sleeve pneumonectomy, standard pneumonectomy, total pneumonectomy, resection of whole lung

Based on the independent risk factors for PMEC prognosis identified through both univariate and multivariate regression analyses—including age, tumor size, N stage, M stage, surgical intervention, and chemotherapy administration—we proceeded to construct a prognostic nomogram aimed at predicting the 1-, 3-, and 5-year OS of PMEC patients(Fig. 4).

**Fig. 5:**
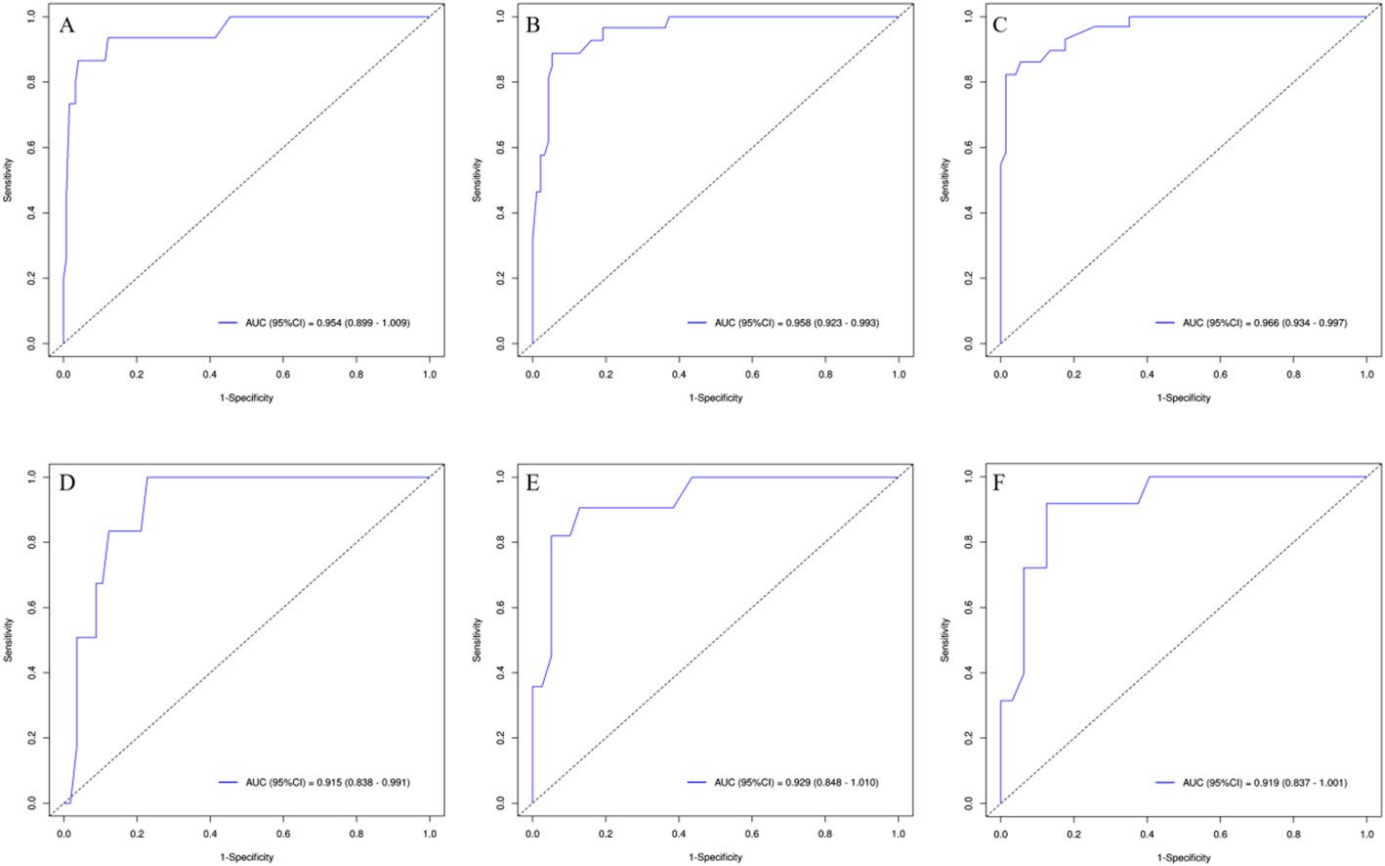
ROC curves for for predicting PMEC patients’OS at 1-year, 2-year, and 3-year for the training (**A–C**), validation (**D–F**)cohorts. *ROC* receiver operating characteristic, *AUC* area under the curve, *OS* overall survival

**Fig. 6:**
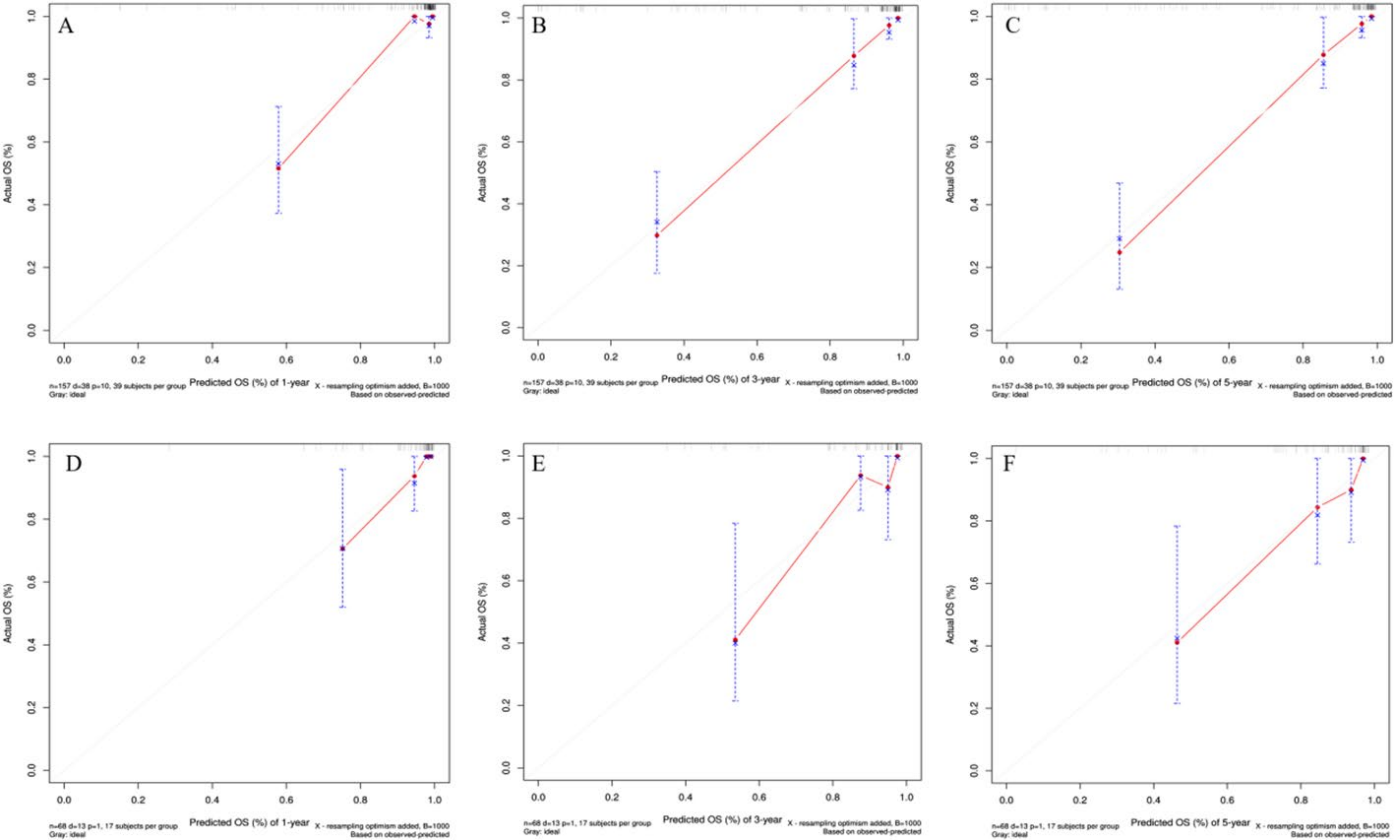
Calibration curves for predicting PMEC patients’ OS at 1-year, 2-year, and 3-year for the training (**A–C**), validation (**D–F**)cohorts. *OS* overall survival

**Fig. 7:**
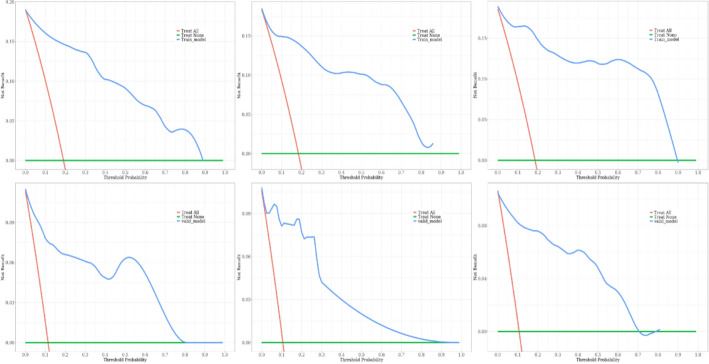
The decision curve analysis of the nomogram for predicting PMEC patients’ OS at 1-year, 2-year, and 3-year for the training (**A–C**), validation (**D–F**) cohorts. *OS* overall survival

To evaluate the performance of the prognostic nomogram for predicting OS in PMEC patients, ROC curves, AUCs, calibration curves, and DCA were employed. Notably, in the testing cohort, the time-dependent ROC curves for 1-year, 3-year, and 5-year OS demonstrated remarkable AUC values of 0.954, 0.958, and 0.966, respectively, indicating a high discriminatory power of the nomogram (Fig. 5). Encouragingly, similar robustness was observed in the validation cohort, with AUCs of 0.915, 0.929, and 0.919 for the same survival endpoints, affirming the consistent predictive accuracy across different datasets. These findings robustly attest to the significant and stable discriminative capability of the constructed nomogram in forecasting survival outcomes for PMEC patients. Further reinforcement comes from the calibration curves, which displayed a close alignment between the predicted probabilities and the actual observed outcomes, thereby reinforcing the model’s precision and reliability (Fig. 6). Additionally, through DCA, we explored the clinical applicability of this model in estimating the OS of PMEC patients. The decision curves illustrated that the nomogram holds substantial clinical utility in improving the prediction of patients’ OS, demonstrating its practical value in guiding therapeutic decisions and risk stratification (Fig. 7). Collectively, these analyses provide compelling evidence for the efficacy, reliability, and clinical relevance of the developed nomogram in predicting the prognosis of PMEC patients.

### Prognosis of PMEC patients with different surgical approaches

**Fig. 8:**
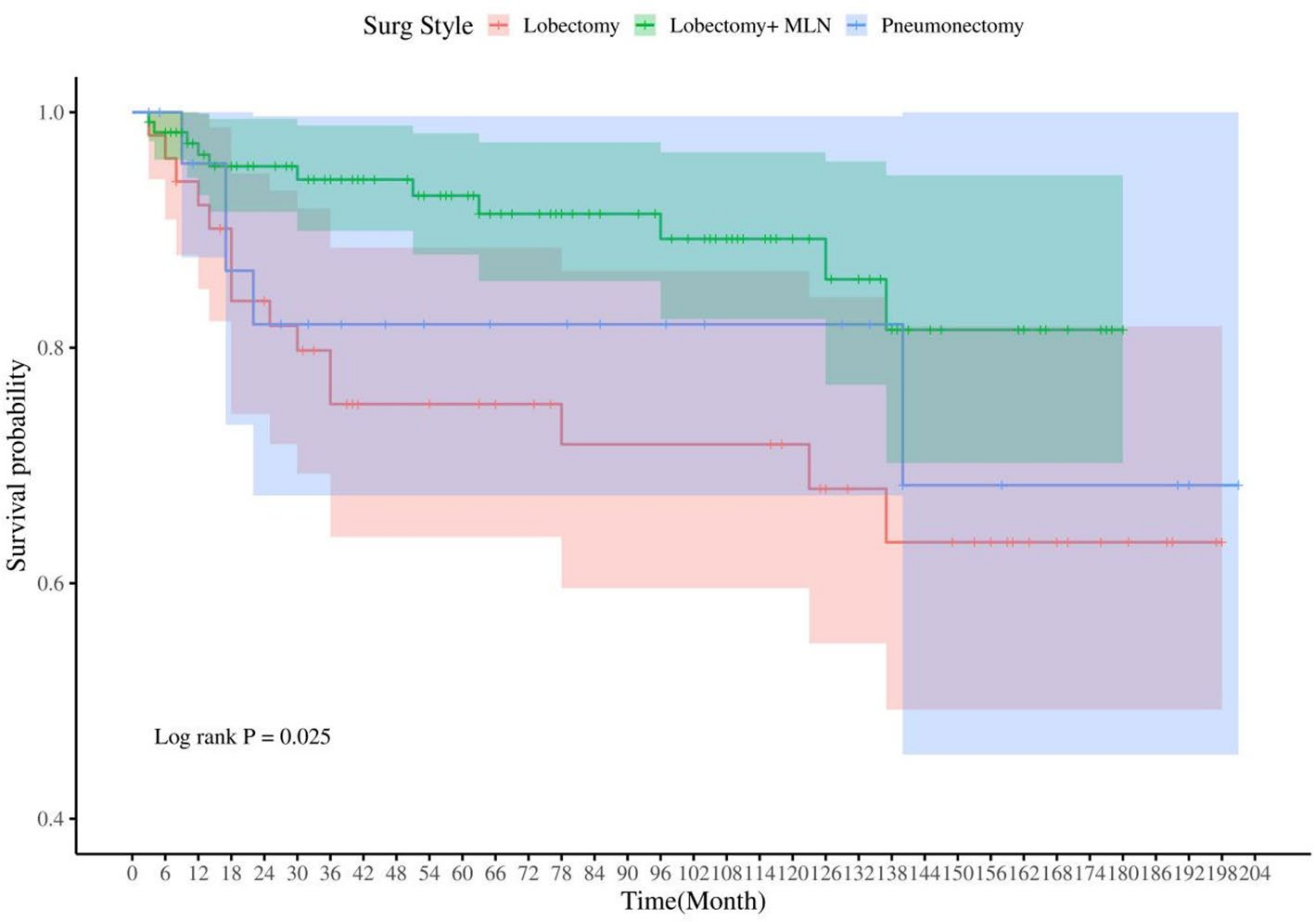
The influence of different surgical methods on the prognosis of PMEC patients.Lobectomy + MLN: Lobectomy WITH mediastinal lymph node dissection; Pneumonectomy: complete pneumonectomy、sleeve pneumonectomy, standard pneumonectomy、total pneumonectomy、 resection of whole lung

To further elucidate the impact of surgical methods on the prognosis of PMEC patients, we conducted Kaplan-Meier (KM) survival analysis. Our findings revealed that patients who underwent “Lobectomy with mediastinal lymph node dissection” exhibited the most favorable prognosis, followed by those who received pneumonectomy (Fig. 8).

## Discussion

PMEC, representing an exceedingly rare form of primary salivary gland-type lung cancer accounting for only 0.1-0.2% of all pulmonary malignancies [2, 3], poses challenges due to its scarcity and limited research. Consequently, its management often resorts to general principles of lung cancer therapy, lacking tailored guidance and prognostic evaluation tools. This study, grounded in the SEER database, aims to explore the epidemiological characteristics and prognostic risk factors of PMEC. Literature reports a wide age spectrum for PMEC incidence, ranging from 3 to 78 years, with a peak incidence in young to middle-aged adults, particularly in the 30–40 age bracket, and a predilection for central airways, often presenting with a more benign course [19]. Similarly In this study we found that 60–69 years (18.7%), 30–39 years (16.44%), and 40–49 years (16%) were the common age groups for PMEC. Consistent with Travis WD et al.‘s observations, 52.89% of our PMEC cases presented tumors measuring 30–52 mm, which are gray-white and friable on histology [20]. PMEC patients generally fare better than those with typical lung cancer, with a 5-year OS rate ranging from 45-70% [21–23]. Our investigation further reveals that PMEC demonstrates superior early survival compared to common lung cancers, with stage I-II patients reaching a 5-year OS of 90.9%. Despite its indolent nature, PMEC eventually progresses to local infiltration and lymph node metastasis [24], leading to a 3-year OS of 23.1% for stage III patients with a median survival of 17 months, and 18.5% for stage IV patients with a median survival of 8.5 months. Therefore, identifying the specific risk factors influencing PMEC prognosis is of utmost importance. Liu Y and colleagues’ study revealed that patient age, lesion location, and disease malignancy are associated with PMEC outcomes [25]. Meanwhile, Cheng D et al. demonstrated that TNM staging and histopathological grading have significant impacts on PMEC [26]. Leveraging the SEER database, we incorporated variables such as age, gender, ethnicity, tumor location, tumor laterality, tumor size, pathologic staging, TNM classification, and treatment modalities (surgery, radiotherapy, chemotherapy) into univariate and multivariate regression analyses to pinpoint the independent factors affecting OS in PMEC patients. Ultimately, we established age,, tumor size, N stage, M stage, surgical intervention, and chemotherapy administration as independent prognostic factors for OS in PMEC patients, akin to the conclusions drawn by Qiu et al. [10], who determined that age above 60, poor differentiation, tumor size exceeding 30 mm, lymph node involvement, and distant metastasis are independently indicative of unfavorable prognosis.

In terms of treatment, while there is currently no standardized therapeutic regimen specifically for PMEC, both domestic and international literature concur on the fundamental principle that surgical intervention is crucial for achieving long-term survival in PMEC patients. For those with advanced disease unsuitable for surgery, treatment strategies resemble those employed for other non-small cell lung cancers (NSCLCs) [27]. Our study delves deeper into the impact of specific surgical approaches on PMEC prognosis, revealing that 52.89% of PMEC patients underwent a combination of lobectomy with mediastinal lymph node dissection, with pneumonectomy and lobectomy being the next preferred options. Survival analyses underscored the superiority of the “lobectomy with mediastinal lymph node dissection” approach for the best patient outcomes, followed by pneumonectomy. Despite previous research suggesting that the efficacy of chemotherapy or radiotherapy as definitive therapies remained unconfirmed [13], our study has identified chemotherapy as an independent factor influencing the prognosis of PMEC patients. In a case report by Sonobe and colleagues, they documented a case of PMEC treated with carboplatin in combination with paclitaxel, where the patient underwent four cycles of this chemotherapy regimen, resulting in significant tumor shrinkage [28]. Additionally, there are several case reports indicating that epidermal growth factor receptor (EGFR) inhibitors, such as gefitinib and erlotinib, have shown efficacy in treating PMEC patients [14, 15, 29, 30]. These insights not only reinforce the primacy of surgery in PMEC management but also underscore the evolving role of adjuvant therapies, particularly chemotherapy and targeted agents, in improving patient outcomes. They emphasize the need for further exploration and personalization of therapeutic strategies to optimize treatment for this rare and complex malignancy.

In this study, we developed and validated a surgical strategy-integrated nomogram for predicting 1-, 3-, and 5-year overall survival (OS) in patients with primary pulmonary mucoepidermoid carcinoma (PMEC). Our findings highlight that age ≥ 60 years, tumor size (30–52 mm), lymph node metastasis, distant metastasis, and the absence of surgical intervention or chemotherapy are independent prognostic factors. Notably, “lobectomy combined with mediastinal lymph node dissection” was associated with the most favorable outcomes, emphasizing the critical role of surgical precision in PMEC management. The nomogram demonstrated robust predictive accuracy (AUC > 0.9) and clinical utility through rigorous internal validation, offering clinicians a practical tool for individualized risk stratification and therapeutic decision-making.

However, our study has limitations. while the SEER database represents the largest available cohort for this rare malignancy, the predominantly White population may limit the generalizability of our findings to other ethnic groups. Second, the retrospective nature of SEER data precluded analysis of potentially important clinical factors including molecular markers (e.g., EGFR/KRAS mutations, CRTC1-MAML2 fusion status), detailed chemotherapy regimens, surgical margin status, and patient comorbidities. Future studies should integrate molecular profiling with clinical variables to develop more precise prognostic models and guide targeted therapies. Third, while internal validation showed high performance, external validation through multicenter cohorts is imperative to confirm its universal applicability.

Future studies should prioritize integrating molecular profiling with clinical variables to refine prognostic models and explore targeted therapies (e.g., EGFR inhibitors) for advanced PMEC. Additionally, international collaborations are encouraged to validate this nomogram in diverse populations and clinical settings. Despite these limitations, our work represents the first prognostic tool incorporating surgical strategies for PMEC, bridging a critical gap in the management of this rare malignancy and paving the way for precision oncology in salivary gland-type lung cancers.

## Conclusion

Our study establishes the first surgical strategy-based nomogram for PMEC, demonstrating exceptional predictive accuracy (AUC > 0.9) and validating lobectomy with lymph node dissection as the optimal surgical approach. This tool addresses a critical unmet need in PMEC management by enabling personalized prognosis and evidence-based surgical decision-making for this rare malignancy.

## Data Availability

Raw data used in analyses is available in SEER database (http://seer.cancer.gov/seerstat).
